# Simulated transient hearing loss improves auditory sensitivity

**DOI:** 10.1038/s41598-021-94429-5

**Published:** 2021-07-20

**Authors:** Patrick Krauss, Konstantin Tziridis

**Affiliations:** 1 Neuroscience Lab, Experimental Otolaryngology, University Hospital Erlangen, Erlangen, Germany; 2 Cognitive Computational Neuroscience Group, University Erlangen-Nürnberg (FAU), Erlangen, Germany; 3 Pattern Recognition Lab, University Erlangen-Nürnberg (FAU), Erlangen, Germany; 4 Department of Otolaryngology, University Medical Center Groningen, Groningen, The Netherlands

**Keywords:** Auditory system, Sensory processing

## Abstract

Recently, it was proposed that a processing principle called *adaptive stochastic resonance* plays a major role in the auditory system, and serves to maintain optimal sensitivity even to highly variable sound pressure levels. As a side effect, in case of reduced auditory input, such as permanent hearing loss or frequency specific deprivation, this mechanism may eventually lead to the perception of phantom sounds like tinnitus or the Zwicker tone illusion. Using computational modeling, the biological plausibility of this processing principle was already demonstrated. Here, we provide experimental results that further support the stochastic resonance model of auditory perception. In particular, Mongolian gerbils were exposed to moderate intensity, non-damaging long-term notched noise, which mimics hearing loss for frequencies within the notch. Remarkably, the animals developed significantly increased sensitivity, i.e. improved hearing thresholds, for the frequency centered within the notch, but not for frequencies outside the notch. In addition, most animals treated with the new paradigm showed identical behavioral signs of phantom sound perception (tinnitus) as animals with acoustic trauma induced tinnitus. In contrast, animals treated with broadband noise as a control condition did not show any significant threshold change, nor behavioral signs of phantom sound perception.

## Introduction

Whether listening to nearly inaudible quiet whispering or enjoying a loud rock concert, the human auditory system has a remarkable ability to adapt to changing sound pressure levels covering several orders of magnitude from the absolute threshold of hearing to the threshold of pain and beyond. Several studies even demonstrate—under certain conditions—the healthy auditory system's ability to further improve sensitivity even below the absolute threshold of hearing^1–4^. However, until recently the underlying neural processes remained rather elusive.

### The stochastic resonance model of auditory perception

In recent studies, we argued that a processing principle called *adaptive stochastic resonance*^5^ is exploited by the auditory system in order to continuously maintain optimal sensitivity even to highly variable sound pressure levels and changing statistics of the acoustic environment^6,7^.

The term stochastic resonance refers to a phenomenon, where a signal of arbitrary kind, which is too weak for a certain sensor for being detected, can be made detectable by adding a random signal, i.e. noise, of appropriate intensity to the sensor input^8,9^. In the last decades, stochastic resonance has been found in a vast number of different organisms and biological systems^10,11^. In particular, in neuroscience stochastic resonance helps to explain how nervous systems robustly operate in noisy environments^12^.

According to our model, stochastic resonance is a major processing principle of the auditory system, and most probably takes place in the dorsal cochlear nucleus^6^. There, auditory input from the cochlea converges with projections from the somatosensory system^13^. Since this somatosensory input is largely uncorrelated with the auditory signals, we argued that these somatosensory projections serve as a random neuronal signal, i.e. neuronal noise, which is necessary for stochastic resonance to work^7^. The intensity of the neuronal noise in the healthy and impaired auditory system is continuously adjusted, depending on the statistics of the auditory input. In case of reduced auditory input for instance, the internal neuronal noise would be upregulated, i.e. somatosensory projections dis-inhibited, which results in increased sensitivity by means of stochastic resonance, thereby enhancing information transmission from the cochlea to the central auditory system. This assumption is supported by empirical findings that somatosensory projections to the cochlear nucleus are actually upregulated after unilateral deafness^14–16^.

### Evidence for stochastic resonance in the auditory system

In neural network simulations, in particular within 3-neuron motifs^17^, we were able to demonstrate that noise even enhances the information flux in such small neural systems, a phenomenon for which the term “recurrence resonance” has been coined^18^. By constructing a hybrid computational model based on deep neural networks, we further demonstrated the biological plausibility of the proposed processing principle, and demonstrated in a simulation of the impaired auditory system that stochastic resonance may even improve, i.e. partly restore, speech recognition after hearing loss^19^. According to our model, the increased internal neuronal noise, i.e. upregulated somatosensory input to the auditory system, corresponds to the observed permanent increase of spontaneous firing rates within the auditory system in case of chronic hearing loss^6^. This neural hyperactivity in turn, was found to be correlated with subjective tinnitus in many studies. Our model provides a unified mechanistic explanation of how hearing loss, phantom perceptions like tinnitus or the Zwicker tone illusion^20^, and neural hyperactivity are related to each other^21^, namely that these phantom perceptions are side effects of the physiological neuronal mechanism to (partly) restore hearing thresholds. Therefore, our model provides a mechanistic explanation why patients with hearing loss and with tinnitus on average have better hearing thresholds than patients with hearing loss and without tinnitus^22^, i.e. why auditory sensitivity is enhanced in tinnitus subjects compared with non-tinnitus subjects^23^. In addition, our model also provides a mechanistic explanation why during the Zwicker tone sensation, auditory sensitivity for tone pulses at frequencies adjacent to the Zwicker tone are improved by up to 13 dB^3^. Tinnitus and Zwicker tone correspond to the perception of the increased neural noise from the somatosensory system, and this noise helps to increase auditory sensitivity by means of stochastic resonance.

Also, work from other groups, that found that not only internal neuronal noise but also external acoustic stimulation with spectral fitted noise stimuli can improve hearing in healthy subjects even beyond the absolute threshold of hearing^1,4^ fits well into our model. It shows that it is not the source of the modulating noise but the spectral information within the auditory system is the important factor for hearing threshold improvement.

Recently, all these implications of our model even lead us to a novel treatment strategy for tinnitus: we already demonstrated in a pilot study, how the presentation of external, spectrally matched, near-threshold acoustic noise, may significantly decrease tinnitus perception by replacing internal neural noise^24^.

### Novel experimental paradigm to test predictions of the stochastic resonance model

In order to provide further evidence for the hypothesis that adaptive stochastic resonance plays a major role in the auditory system and especially in phantom sound perception, we developed a novel animal experimental paradigm: *simulated hearing loss through long-term noise exposure with notched noise* at moderate, non-damaging sound intensities. This paradigm provides the possibility to simulate transient hearing loss and complete recovery from hearing loss without changing the peripheral or central auditory system permanently, e.g. sound trauma induced cochlear damage.

In nature, the standard acoustic environment, i.e. the temporally averaged frequency power spectrum, is broad and flat, i.e. all frequencies have statistically similar mean sound pressure levels—at least on time scales of days to weeks, and at least in the frequency range best perceivable by animals and humans^25^. Therefore, it seems possible to simulate a certain hearing loss by exposure with sound or noise where the mean (i.e. temporally averaged) power of the frequency band of desired hearing loss is decreased (= dipped) compared to adjacent frequencies. Hence, the otherwise flat power spectrum has a dip that mimics hearing loss. From the perspective of the auditory system, it should make no difference whether certain frequency channels receive less input due to cochlear damage or due to changed statistics of the acoustic environment. After the end of long-term acoustic noise exposure, animals' acoustic environment has again a flat frequency power spectrum on longer time scales, which, analogously to the above argument, corresponds to a complete restoration of hearing. Thus, the post-noise exposure phase of the paradigm corresponds to a complete restoration of hearing, or a simulated treatment with an ideal hearing aid, respectively.

## Results

**Figure 1:**
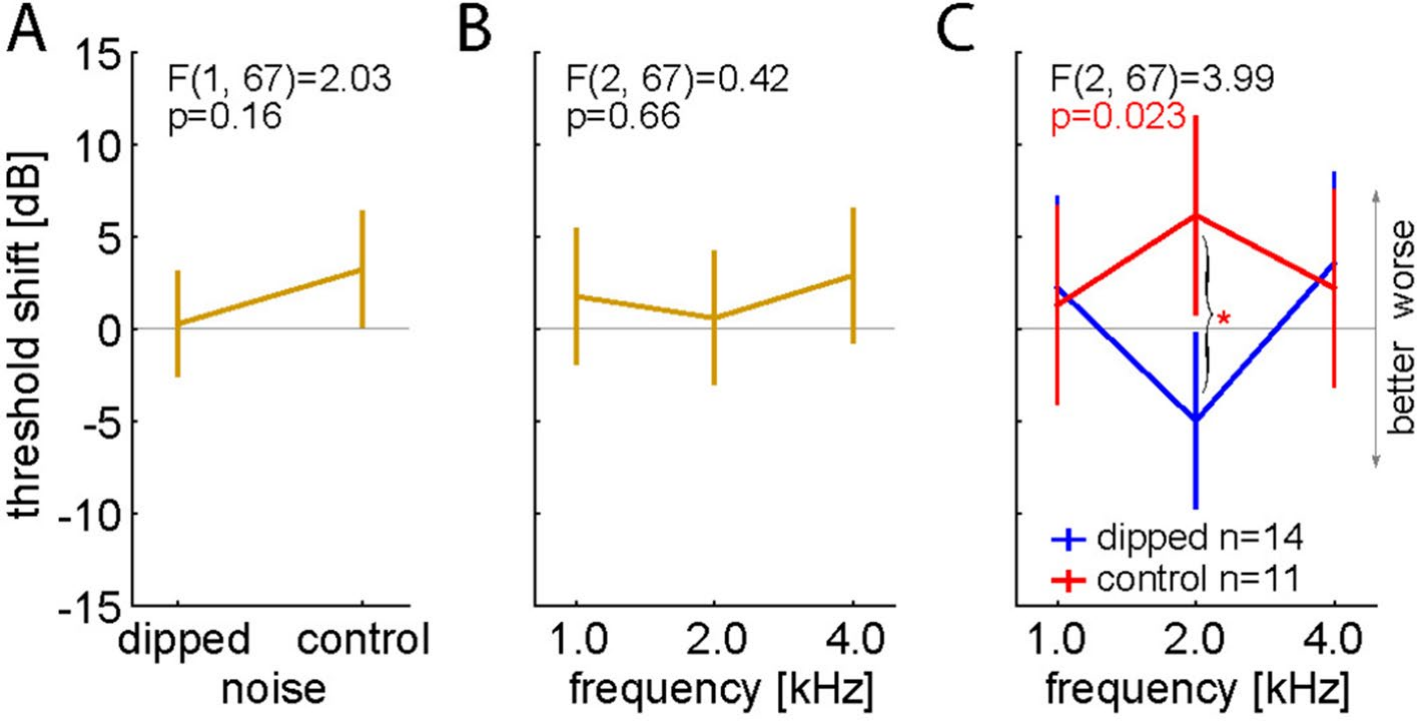
Long-term dipped noise exposure induced improvement of hearing thresholds. Two-factorial ANOVA results of hearing threshold shifts with factors noise and frequency. (**A**, **B**) Non-significant one-factorial analysis parts of noise (**A**) and frequency (**B**). (**C**) Significant interaction of both factors. Animals treated with 2 kHz spectral dipped noise (n = 14) show a significant (p = 0.028, Tukey post-hoc test) mean decrease (= improvement) of hearing thresholds at 2 kHz compared to control (n = 11) animals’ hearing thresholds. The hearing thresholds for 1 and 4 kHz (flat spectrum) do not show significant differences between the groups. Error bars indicate 95% confidence intervals.

Fourteen animals (Mongolian gerbils) have been treated with the new paradigm (2 kHz spectral dipped noise), and additional 11 animals with the control paradigm (spectral flat noise). The relative mean threshold changes immediately after treatment are summarized in Fig. 1 by two-factorial ANOVA plots (Factors: *noise group* and *frequency*; F-statistics given there). Neither in the factor *noise group* (Fig. 1A), nor in the *frequency* domain (Fig. 1B) any significant effects could be found. But in the interaction of both factors, animals treated with 2 kHz spectral dipped noise showed a significant (p = 0.028, Tukey post-hoc test) mean decrease (= improvement) of hearing thresholds at 2 kHz compared to the control animals’ threshold shifts (Fig. 1C, blue). Additionally, only at the 2 kHz testing frequency, these treated animals showed a significantly (p = 0.01, Bonferroni corrected single sided t-test vs. 0) decreased mean threshold shift from zero (Table 1).

**Table 1:** Results of Bonferroni corrected single sided t-tests of mean threshold shifts against zero.

Noise	Frequency (kHz)	Mean threshold shift (dB)	Standard deviation	Corrected p value
Dipped	1.0	2.23	12.57	1.0
	2.0	− 4.98	5.59	0.01
	4.0	3.57	6.99	0.18
Control	1.0	1.29	4.59	0.75
	2.0	6.16	12.86	0.29
	4.0	2.20	6.40	0.56

In contrast, hearing thresholds for 1 and 4 kHz (where the spectrum was flat) did not change significantly (Fig. 1C, red). The control group stimulated with spectral flat noise showed no significant shift in any tested frequency (Table 1). These results are perfectly in line with the hypothesis that the auditory system exploits stochastic resonance to compensate for changing input statistics.

**Figure 2:**
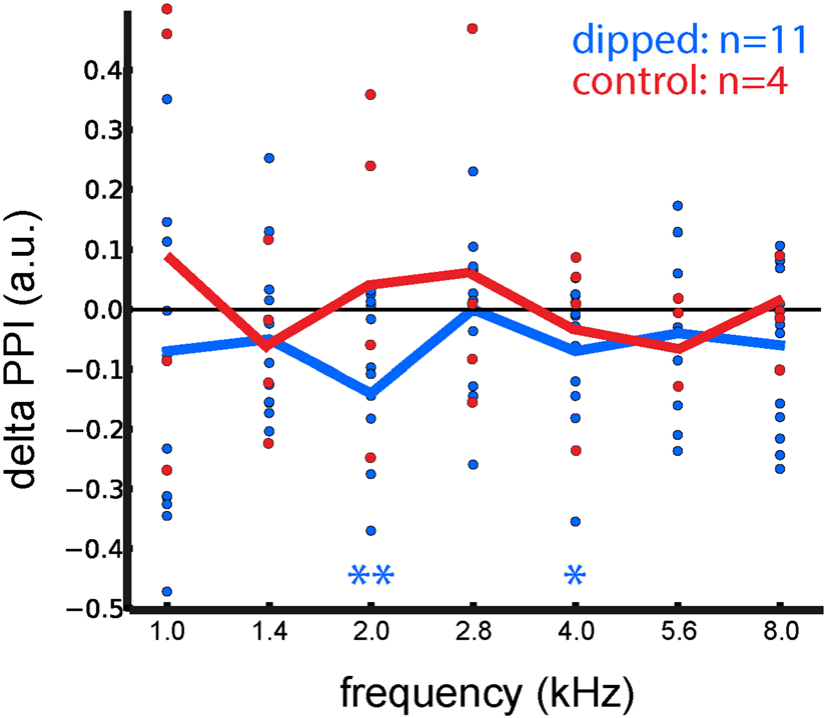
Acute behavioral signs of tinnitus. Immediately after long-term notched noise exposure, mean ∆PPI values (blue) are significantly negative at 2 and 4 kHz (2 kHz: p < 0.01, t value − 3.11519, df 11; 4 kHz: p < 0.05, t-value − 2.27799, df 10; two-sided paired t-test, Bonferroni corrected for multiple testing). In contrast, PPI values of the control animals (red) do not change significantly.

In addition, most tested animals (cf. “Methods”; 10 of 11 tested animals) treated with the new paradigm showed identical behavioral signs of phantom sound perception as animals with acoustic trauma induced tinnitus, whereby the strongest acute effects occurred at the frequency of 2 kHz where the spectral dip was centered (Fig. 2). In the control group, no statistically significant behavioral signs of phantom sound perception could be observed (Fig. 2). Again, this supports our stochastic resonance model of tinnitus development. Only one animal did not show any behavioral signs of tinnitus, similar to the observed ratio of tinnitus development after noise trauma.

**Figure 3:**
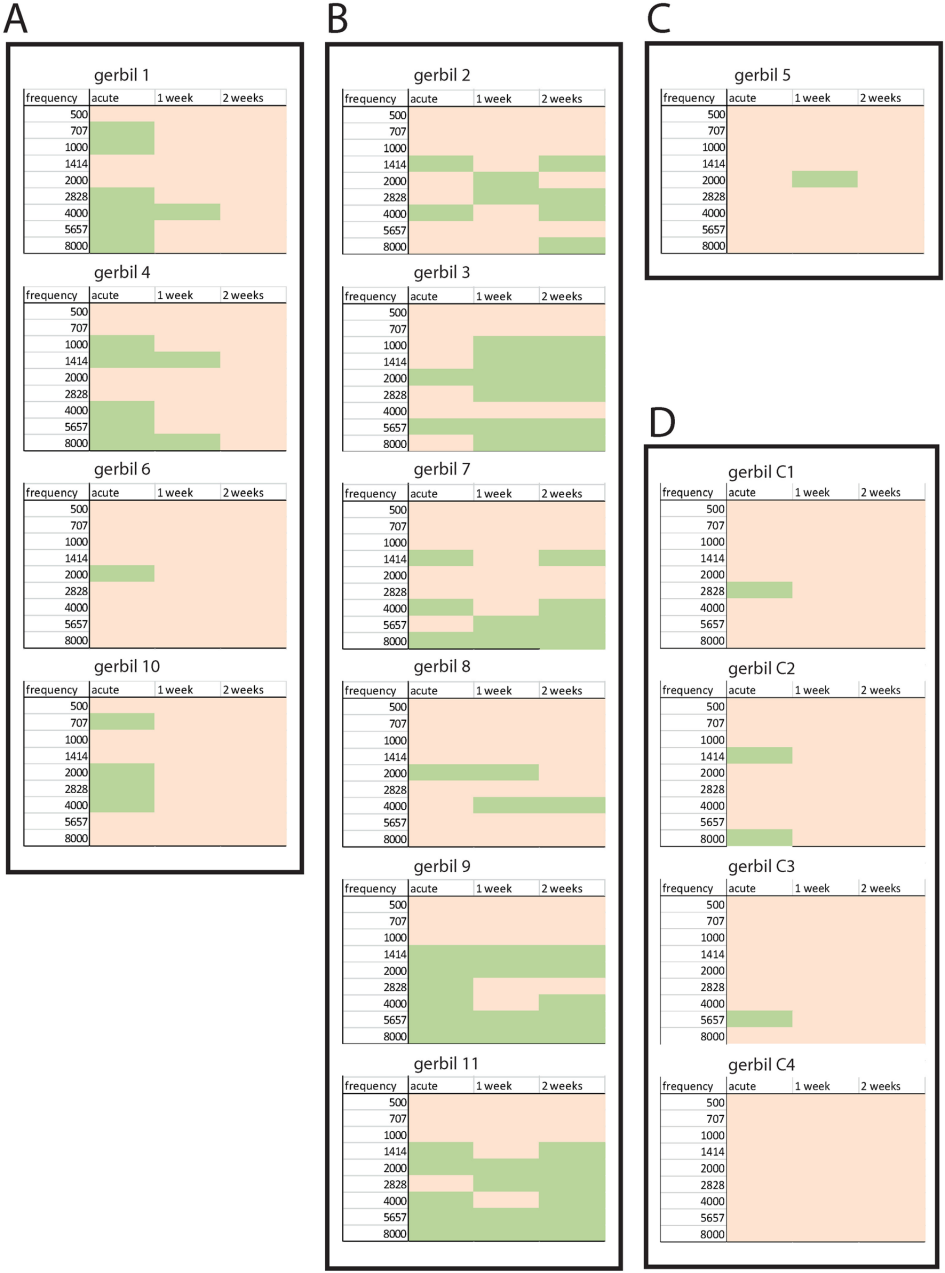
Temporal development of behavioral signs of tinnitus. (**A**, **B**) Immediately after notched noise exposure 10 of 11 tested animals showed behavioral signs of tinnitus (green fields). (**C**) Only one animal treated with notched noise did not develop tinnitus. Within 2 weeks after notched noise exposure tinnitus related behavioral signs vanished completely in four animals (**A**). In six animals, tinnitus was not removed but became chronic instead (**B**). (**D**) In the control group, three of four tested animals showed few behavioral signs of tinnitus only immediately after treatment with broadband noise. Green fields indicate behavioral signs of tinnitus, defined as significant changes of the effect size of the PPI change. Pink fields indicate non-significant changes. Note that, on a 5% level we cannot rule out that we detect false positive behavioral signs of tinnitus in a certain frequency range. In principle, also gerbil 6 could be assigned to group C. However, since in gerbil 6, we detect the behavioral signs of tinnitus immediately after instead of 1 week after treatment as it is the case in gerbil 5, it is therefore more likely that gerbil 6 is a true positive while gerbil 5 is a false positive.

After the end of long-term notched noise exposure which corresponds to subsequent simulated treatment with a perfect hearing aid, one group of animals (n = 4) showed a complete removal of behavioral signs of tinnitus (Fig. 3A), whereas another group did not respond to the end of the treatment. In those animals (n = 6) the tinnitus perception became chronical and could be observed even 2 weeks after the end of notched noise exposure (Fig. 3B). A further animal did not show any behavioral signs of tinnitus immediately after notched noise exposure (Fig. 3C). Remarkably, these results correspond to what is known from tinnitus patients treated with hearing aids or cochlea implants. In one group of patients, the tinnitus perception is reduced or even completely removed, whereas another group does not respond to the treatment^26–28^. In the control group, three of four tested animals showed few behavioral signs of tinnitus only immediately after treatment with broadband noise (Fig. 3D).

Interestingly, the animals that developed behavioral signs of tinnitus during notched noise exposure can be divided into two groups: a broadband tinnitus group, where more than two frequencies are affected (n = 5), and tonal tinnitus group, where only one or two frequencies are affected (n = 4). In the tonal tinnitus group, three of the four animals show behavioral signs of tinnitus exactly where expected, namely at 2 kHz. However, why either broadband tinnitus or tonal tinnitus develops during notched noise exposure remains unclear and may be part of further studies.

## Discussion

We presented a novel experimental paradigm to simulate transient hearing loss, which in turn induced improvement of hearing thresholds and perception of phantom sounds when hearing was normal again. Both effects are in line with our hypothesis that stochastic resonance plays a major role in the auditory system. The converging evidence from numerous empirical (for an overview see^7,21^) and theoretical studies^5,6,19^ indicates that the auditory system actually exploits stochastic resonance and actively tunes the intensity of the required neuronal noise by adjusting the somatosensory input to the dorsal cochlear nucleus. By that, stochastic resonance contributes crucially to the auditory system’s ability to adapt to changing sound pressure levels.

It is important to mention that hearing was not impaired permanently in the treated animals. The end of the sound exposure corresponds to the end of the simulated hearing impairment and therefore threshold changes could be only observed directly after the end of the stimulation in the then healthy auditory system. In terms of time scale, the induced phantom sounds—as a side effect of the physiological mechanism to compensate for reduced auditory input—are at an intermediate level between chronic lifelong tinnitus on the one end and the only seconds lasting Zwicker tone illusions^20^ on the other end of the spectrum. Within the stochastic resonance framework of auditory processing, all these phantom sounds are caused by the same neuronal mechanism and do just occur on different time scales^21^.

Furthermore, the presented results are similar to ear plugging studies, where healthy human subjects are provided with ear plugs for 2 weeks, mimicking hearing loss. These subjects perceive a transient tinnitus after removal of ear plugs^29,30^. In contrast to ear plugging, where mainly higher frequencies are attenuated, the here presented long-term notched-noise exposure paradigm has the advantage that both, spectral location and shape of simulated hearing loss are fully controllable. In follow-up studies, it might be interesting to systematically compare different shapes of hearing loss, e.g. high-frequency hearing loss versus low-frequency hearing loss. Furthermore, the impact of other kinds of noise (e.g. babble noise or correlated noise) on hearing threshold changes and induction of phantom sounds may be investigated.

It is worth mentioning that in ear plugging studies, no subject reported tinnitus 2 weeks after the end of ear plugging, which seems to be in contrast with the here presented results. However, we speculate that this has something to do with the fact that neural processing and homeostatic plasticity in rodents takes place on shorter time scales than in humans. For instance the maximum duration of the Mongolian gerbil’s auditory sensory memory for random waveforms is about 80 ms^31^, whereas in humans the corresponding duration is up to 20 s^32^. Thus, 2 weeks of notched noise exposure in rodents correspond to a much longer treatment with ear plugs in humans than just 2 weeks; this may also explain the large difference of more than 11 dB threshold shift between the treated and control group. Hence, one might hypothesize that, if humans would be treated with ear plugs for a longer time, then the tinnitus sensation may become chronic in some subjects as well. This would indicate that the duration of ear plugging or notched noise exposure correlates with the ratio of subjects or rodents developing chronic tinnitus. This is an interesting question to be addressed—at least on the level of rodents—in a follow-up study.

Furthermore, one may argue that, since thresholds obtained with sensitive auditory brainstem response (ABR) methods are in most animals approximately 10–20 dB above neuronal or behavioral data, the measured responses do not necessarily reflect threshold sensitivity, but rather some nonlinear interaction of a larger number of neurons, which makes it hard to interpret in terms of sensitivity. To decide this question, a replication of the presented study could be performed, yet with the difference that hearing thresholds are estimate with some behavioral paradigm, or cortical recordings, instead of ABR measurements.

As mentioned in the results the animals develop either broadband or tonal tinnitus, which does not seem to correlate with either being transient (Fig. 3, group A) or permanent (Fig. 3, group B). Why an individual animal develops either the one or the other form of tinnitus and why it recovers in one case and not in the other remains elusive and needs further investigation in a larger sample.

The clinical relevance of our study is underlined by the fact that recently, the implications of our model lead us to a novel treatment strategy for tinnitus: we already demonstrated in a pilot study, how the presentation of external, spectrally matched, near-threshold acoustic noise, may significantly decrease tinnitus perception^24^. Finally, we note that our paradigm is an important step towards reducing the health burden on laboratory animals, since instead of applying a permanent noise trauma in order to induce tinnitus, the hearing loss can now be simulated.

## Methods

### Animals and ethics statement

Twenty-five Mongolian gerbils (*Meriones unguiculatus,* male, 12 weeks old) were housed in standard animal racks (Bio A.S. Vent Light, Zoonlab GmbH, Castrop-Rauxel, Germany) in groups of two animals per cage with free access to water and food at 20–24 °C room temperature under 12/12 h dark/light cycle. The use and care of animals was approved by the state of Bavaria (Regierungspräsidium Mittelfranken, Ansbach, Germany; AZ: 54-2532.1-02/13 and 54-2532.1-42/13). All methods were carried out in accordance with relevant guidelines and regulations. Furthermore, the study was carried out in compliance with the ARRIVE guidelines. Gerbils were purchased from Charles River Laboratories Inc. (Sulzfeld, Germany).

### Generation of noise stimuli

Starting from white noise with flat frequency spectrum, dipped noise has been generated with equalizer software. The dip has a spectral width of an octave centered on a frequency of 2 kHz. At the center frequency the power-difference to the non-dipped (= flat) spectrum is maximal (20 dB) and decreases sigmoidally (in order to avoid sharp edges in the spectrum) with increasing distance to the center. At the borders (1.4 and 2.8 kHz) the power-difference is zero, i.e. the power is identical to the rest of the power spectrum. For the control group non-dipped (= flat) white noise was used.

### Long-term noise exposure and experimental protocol

Exposure to 2 kHz dipped (n = 14)/flat (n = 11) noise at low sound pressure levels (50 dB SPL) took 2 weeks for each animal. The notched noise had a dipped spectral envelope. This new paradigm simulates both hearing loss (dip in frequency spectrum) and potential subsequent treatment with an ideal hearing aid or therapy that fully restores hearing abilities. Hearing thresholds for frequencies of 1, 2 and 4 kHz were measured using ABR in all animals before and immediately after long-term noise exposure. The GPIAS paradigm^33,34^ was used to assess the potential existence of a tinnitus percept. Animals were tested before (pre), immediately after (acute), 1 week and 2 weeks after long-term noise exposure.

### Assessment of hearing thresholds

In order to assess the animal's hearing thresholds, ABR were recorded using a custom made setup^35^. Pure tone stimuli of different frequencies (1.0, 2.0 and 4.0 kHz) were generated by a custom-made python program and presented at different, pseudo-randomized intensities ranging from 90 to 0 dB SPL in 5 dB steps. Stimulation was free-field to the measured ear (ipsilateral) at a time via a speaker (Sinus Live NEO) corrected for its frequency transfer function to be flat within ± 1 dB at a distance of approximately 30 mm from the animal's pinna while the contra-lateral ear was tamped with an ear plug. To compensate for speaker artifacts stimuli were presented as double trials consisting of two 6 ms stimuli (including 2 ms sine square rise and fall ramps) of the same amplitude but opposite phase, separated by 100 ms of silence. 120 double trials of each combination of intensity and frequency were presented pseudo-randomly at an inter-stimulus interval of 500 ms.

For the measurements the Mongolian gerbils were kept under deep anesthesia. Anesthesia was induced by an initial dose of 0.3 ml of a ketamine-xylacin-mixture (mixture of ketamine hydrochloride: 96 mg/kg BW; xylacin hydrochloride: 4 mg/kg BW; atropine sulfate: 1 mg/kg BW), and maintained by continuous application of that mixture at a rate of 0.2–0.3 ml/h by a syringe pump. As has been demonstrated previously, such ketamine–xylazine anesthesia has only little effect on ABR signals compared to awake animals^36^.

During measurements, animals were placed on a heating pad at 37 °C. Data were recorded using three silver wire electrodes positioned subcutaneously, one for grounding at the back of the animals, one reference electrode at the forehead and the measuring electrode infra-auricular overlying the bulla. The potential difference between the reference and measuring electrode was amplified by a low noise amplifier (JHM NeuroAmp 401, J. Helbig Messtechnik, Mainaschaff, Germany; amplification 10,000; bandpass filter 400–2000 Hz and 50 Hz notch filter). Note that for further analysis the amplified signal was used, that is, amplitudes are given in mV whereas the actual neuronal signals were in µV range. The output signal of the amplifier was digitalized and recorded by an analog–digital converter card (National Instruments Corporation, Austin, TX, USA) with a sampling rate of 20 kHz and synchronized with the stimulation via the trigger signal of the stimulation computer. Raw data of 120 double trials per sound level for one stimulus frequency were averaged. Finally, these averaged responses of the two single, phase inverted stimuli within one double trial were averaged to eliminate stimulus artifacts. From these averaged, artifact-corrected data the root mean square (RMS) values within a time window from 0 to 10 ms after stimulus onset were calculated to obtain a measure of ABR amplitude for each stimulus intensity presented.

Hearing thresholds were estimated by fitting the ABR root-mean-square amplitudes to different stimulus intensities with a hard sigmoid function. In combination with subsampling, this universally applicable procedure provides a robust threshold estimation as well as an accurate uncertainty estimate. Furthermore, this method has no systematic dependence on the noise and does not even require samples in the full dynamic range of the sensory system (for a detailed description of the method see^35^).

### Phantom sound assessment (GPIAS)

Putative perception of phantom sounds was assessed with the gap pre-pulse inhibition of the acoustic startle reflex (GPIAS) paradigm^33^ using a custom made setup^37^. Briefly, the animals were placed in a transparent acrylic tube (length 10 cm, inner diameter 4.3 cm) which was positioned at a distance of 10 cm in front of two loudspeakers, one for the moderate background noise and one for the startle stimulus. The startle response was measured by a sensor platform with three integrated acceleration sensors (ADXL 335 on GY 61 board, Robotpark). Stimulus generation and data acquisition used custom-made software (Python, Version 3.6.0). As startle amplitudes tend to be higher for the first few trials, the first five startle stimuli were presented before the beginning of each measurement to rule out strong habituation effects. All animals were then subjected to continuous band pass filtered 60 dB SPL loud background noise (2 ms cosine square rise and fall times) with medium frequencies of 1–8 kHz in ½ octave steps and a bandwidth of ± ¼ octave. The mean duration of the background noise before the startle noise burst was 10 ± 2.5 s, it ended at the beginning of the startle noise burst. The startle white noise burst (115 dB SPL, 20 ms) was presented either 50 ms after a 50 ms long silent gap (2 ms cosine square rise and fall times) in the background noise, or it was presented without any gap. For each background frequency, 20 repetitions with and without gap were presented in randomized order. Every animal was tested four times, the first time in a naïve condition before treatment, and the other times immediately after, 1 week and 2 weeks after being taken out of the long-term noise exposure. The whole experimental session did not last longer than 30 min.

Data were analyzed using the method described in^34^. Briefly, as the measured response amplitudes are log-normally distributed, acquired amplitudes were logarithmized. Then we exploited the full combinatorial power of all log-normalized response amplitudes to obtain the distributions of the prepulse inhibition (PPI) before and after treatment of the animals. From these distributions, we calculated the change of the behavioral response. Positive values indicate a stronger effect of the gap in the post compared to the pre condition. Negative values indicate less effect of the gap after treatment, i.e., a stronger startle response despite the present gap, which indicates a “filling” of the gap by a tinnitus percept in that frequency range. Additionally, these now normally distributed data were analyzed using parametrical Student’s t-tests to find significant changes of the PPI change (ΔPPI) which can be used to define the strength of a possible tinnitus percept.

Due to experimental constrains, only 11 out of the 14 animals with 2 kHz dipped noise and 4 out of 11 control animals with flat noise exposure could be tested with this paradigm.

### Statistical evaluation

Threshold shifts have been calculated by subtracting the pre exposure ABR threshold from the post exposure ABR threshold at each tested frequency for each individual. By this, a negative threshold shift corresponds to a better hearing after noise exposure at a given frequency. The threshold shifts have been analyzed by a two-factorial ANOVA using Statistica 8 (StatSoft, Hamburg, Germany) with the factors *noise* group and stimulation *frequency*. Post-hoc tests were performed with Tukey tests, already correcting for multiple comparisons. Additionally the threshold shifts at the different stimulation frequencies of both noise exposure groups were tested against zero (= no shift) by single sided t-tests, Bonferroni corrected for multiple comparisons. These tests were undertaken to rule out, that the control animals did show an “inverse” effect compared to the 2 kHz dipped noise exposure group. As mentioned above, the changes in GPIAS responses have been analyzed with a custom made Python 3.6 software described in^34^. The reason for using t-tests (corrected for multiple comparisons) here is the at least partial independency of each tested frequency relative to the other and the reduction of perquisites required for the statistics. We wanted to know, at which frequency exactly a possible tinnitus percept emerged and not only if it is somewhere in the data (as, e.g., with a one-factorial ANOVA).
